# Changes to the Aqueous Humor Proteome during Glaucoma

**DOI:** 10.1371/journal.pone.0165314

**Published:** 2016-10-27

**Authors:** Martha Andrea Kaeslin, Hanspeter Ezriel Killer, Cyril Adrian Fuhrer, Nauke Zeleny, Andreas Robert Huber, Albert Neutzner

**Affiliations:** 1 Center of Laboratory Medicine, Cantonal Hospital Aarau, Tellstrasse 25, 5001 Aarau, Switzerland; 2 Department of Ophthalmology, Cantonal Hospital Aarau, Tellstrasse 25, 5001 Aarau, Switzerland; 3 Department of Biomedicine and Department of Ophthalmology, University Basel, Basel, Switzerland; Massachusetts Eye & Ear Infirmary, Harvard Medical School, UNITED STATES

## Abstract

**Purpose:**

To investigate the aqueous humor proteome in patients with glaucoma and a control group.

**Method:**

Aqueous humor was obtained from five human donors diagnosed with primary open angle glaucoma (POAG) and five age- and sex-matched controls undergoing cataract surgery. Quantitative proteome analysis of the aqueous humor by hyper reaction monitoring mass spectrometry (HRM-MS) based on SWATH technology was performed.

**Results:**

Expression levels of 87 proteins were found to be different between glaucomatous and control aqueous humor. Of the 87 proteins, 34 were significantly upregulated, whereas 53 proteins were downregulated in the aqueous humor from glaucoma patients compared to controls. Differentially expressed proteins were found to be involved in cholesterol-related, inflammatory, metabolic, antioxidant as well as proteolysis-related processes.

**Conclusion:**

Glaucoma leads to profound changes to the aqueous humor proteome consistent with an altered metabolic state, an inflammatory response and impaired antioxidant defense.

## Introduction

Glaucoma is a leading cause for vision loss projected to cause blindness in about 80 million people worldwide by 2020 [1]. Clinically, glaucoma presents with visual field loss and ultimately loss of central vision, resulting from slowly progressing degeneration of retinal ganglion cells and their axons. The latter make up the optic nerve and is responsible for relaying pre-processed signals from the retina to the optic nerve. On fundus examination, glaucoma is characterized by pathological excavation of the optic disc, and a marked loss of supporting glia cells. Several types of glaucoma are known, ranging from the rare congenital and acute angle-closure variants to the most frequent chronic primary open-angle glaucoma (POAG) with either increased or normal intraocular pressure [2, 3]. Elevated intraocular pressure is an established risk-factor for glaucoma. It is caused by an imbalance between the production of aqueous humor and the drainage of this liquid through the trabecular meshwork lining the anterior chamber angle. Another risk factor for glaucoma is vascular dysregulation and associated hypoxic and oxidative stress conditions, which in form of the Flammer syndrome is associated with normal tension glaucoma [4]. In addition to increased intraocular pressure and vascular dysregulation, the optic nerve compartment syndrome was recently connected to glaucoma and other related diseases affecting the function of the optic nerve [5].

How increased intraocular pressure, vascular dysregulation and optic nerve compartmentalization ultimately lead to the death of retinal ganglion cells and the associated damage to the optic nerve is not fully understood.

To understand biochemical changes during glaucoma progression and to identify potential biomarkers, we analyzed the proteome of aqueous humor from glaucoma patients by mass spectrometry and compared it to a control group recruited from patients without glaucoma that were undergoing cataract surgery. We identified a total of 448 proteins in the aqueous humor of glaucoma and control patients with 34 proteins up- and 53 downregulated proteins. Gene ontology (GO) analysis revealed the suppression of metabolism related proteins as well as the upregulation of proteins connected to immunological and protease activity regulating processes.

## Results

**Fig 1:**
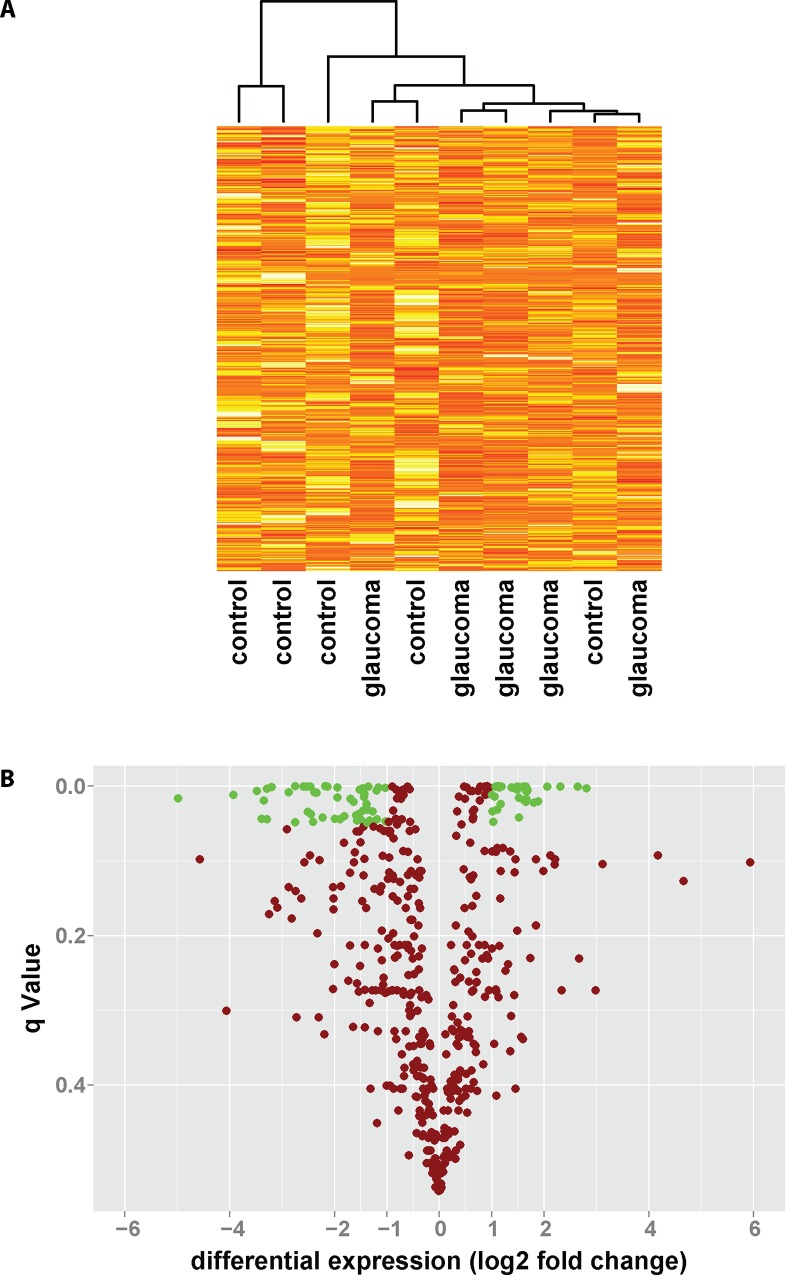
Comparing the aqueous humor proteome of glaucoma patients to controls. The proteome of aqueous humor samples obtained from POAG patients or control subjects undergoing cataract surgery was determined using quantitative hyper reaction monitoring mass spectrometry with SWATH technology. **(A)** Cluster analysis of glaucoma patients and controls based on the expression level of 448 proteins in aqueous humor. **(B)** Of 448 proteins measured, 53 proteins showed significant downregulation, while 34 proteins showed significant upregulation in the aqueous humor of glaucoma patients compared to controls. Shown is the differential expression in log2 fold change against the significance level of the false discovery rate test (q-value).

Quantitative mass spectrometric analysis of aqueous humor from glaucoma patients and controls was performed and a total of 448 proteins were identified and their levels were determined. Cluster analysis of samples based on the identity and amount of protein was performed and revealed separation of 4 out of 5 glaucoma patients from controls (Fig 1A). To further assess differences in the aqueous humor proteome, volcano plot analysis was performed. As shown in Fig 1B, while 53 proteins showed significantly lower concentrations in glaucomatous aqueous humor, levels of 34 proteins were significantly increased compared to controls.

**Fig 2:**
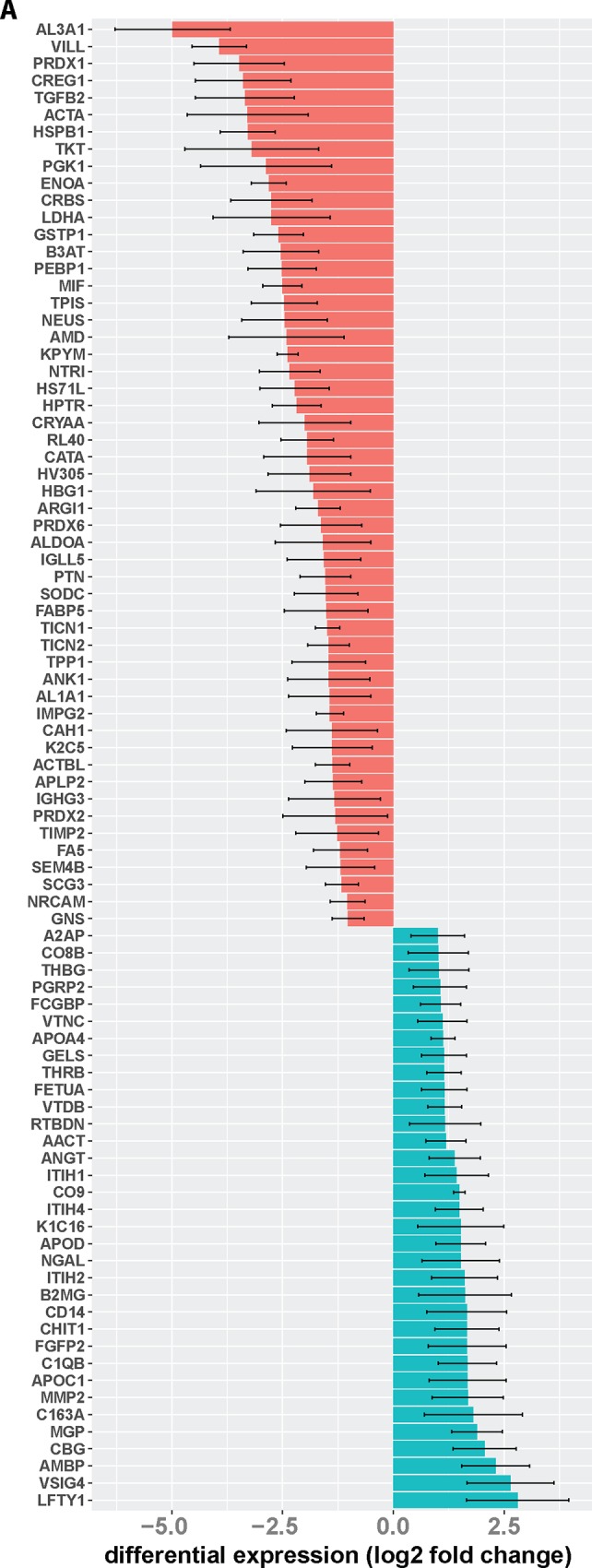
Glaucoma-related changes to the aqueous humor proteome. Shown are gene names for proteins with significantly diminished (red) or increased (green) levels in the aqueous humor of glaucoma patients compared to controls. Differential expression is presented as log2 fold change. Differential regulation was assessed using false discovery rate test and q < 0.05 was considered significant. Error bars represent SEM.

Significant differential expression of proteins in the glaucomatous aqueous humor ranged from an about—97% reduction for aldehyde dehydrogenase (AL3A1) to an about 7-fold increase for Left-Right Determination Factor 1 (LFTY1) (Fig 2A).

**Fig 3:**
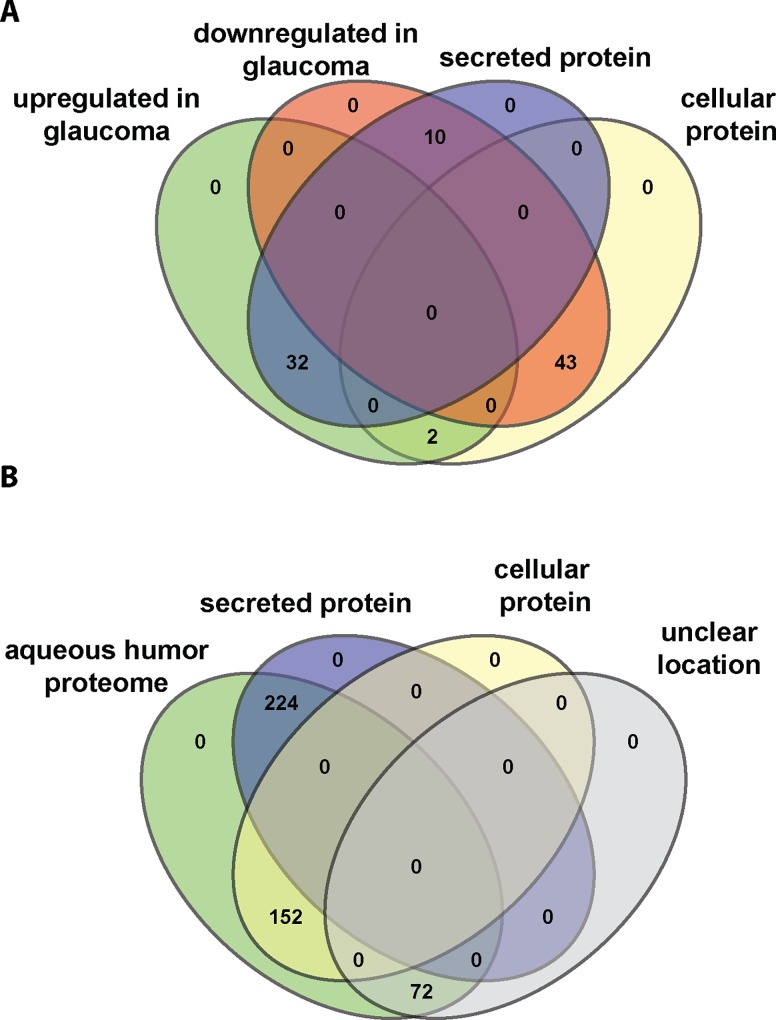
Secreted proteins are enriched in the glaucomatous aqueous humor proteome. **(A)** By querying the Uniprot database, proteins were categorized as secreted or intracellular proteins. Shown is the numbers of proteins either up- or downregulated in glaucoma with their respective location. **(B)** Venn diagram depicting the location (secreted, cellular, unclear) of all proteins identified in the aqueous humor.

The aqueous humor is secreted by the ciliary body and its proteome is expected to be enriched in secreted proteins. Therefore, we analyzed differentially expressed proteins in terms of their localization and categorized them into secreted, (intra)cellular, as well as protein of unknown location. Interestingly, out of 34 proteins upregulated in glaucoma 32 are secreted while 2 are intracellular proteins. In contrast, downregulated proteins were mostly intracellular with 10 out of 53 proteins found to be secreted (Fig 3A). When considering the aqueous humor protein as determined by us (Fig 3B), 224 out of 448 proteins were secreted, while 152 proteins are intracellular proteins and 72 proteins are annotated with unclear localization. Thus, upregulated proteins in glaucoma are enriched for secreted proteins compared to controls and the entire aqueous humor proteome, while intracellular proteins are overrepresented among proteins downregulated in glaucoma.

**Fig 4:**
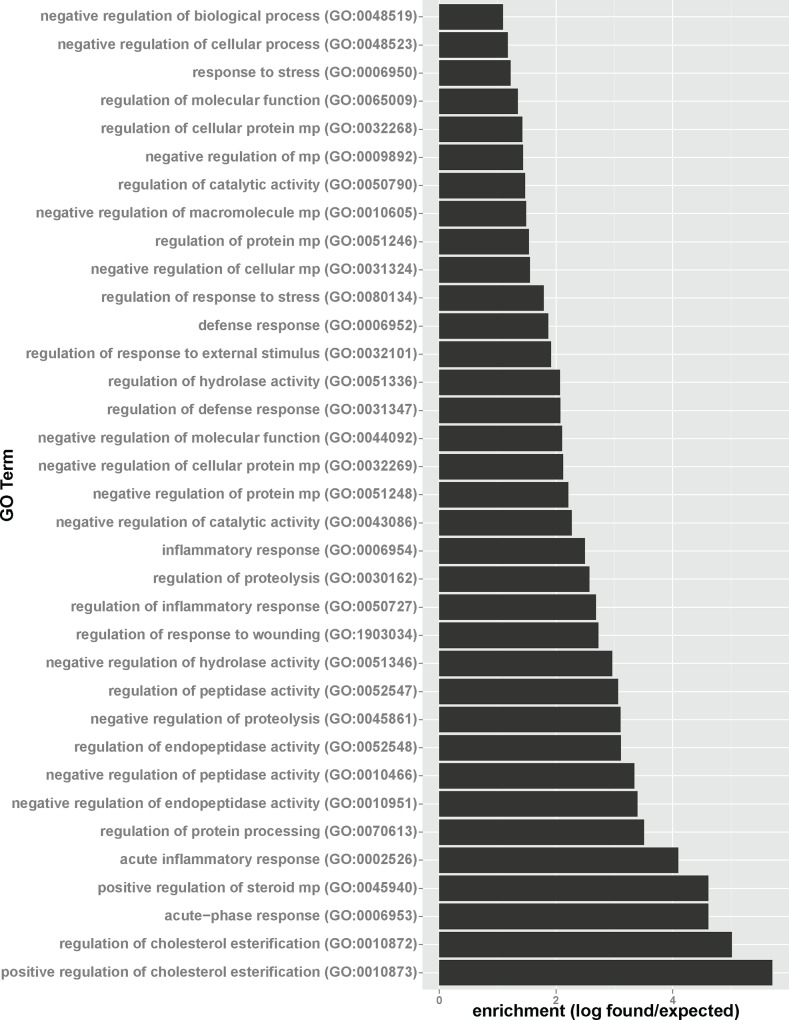
Glaucoma-related upregulation of proteins involved in cholesterol metabolism, endopeptidase regulation, stress and immunological response. Proteins found to be upregulated in glaucomatous aqueous humor were analyzed for GO term enrichment. Shown are significantly enriched GO terms (p-Value < 0.01 with Bonferroni correction for multiple comparisons) with enrichment defined as number of proteins found/number of proteins expected based on the human reference genome.

To decipher biochemical pathways in the aqueous humor significantly impacted by glaucoma, gene ontology analyses for up- and downregulated proteins were performed. For proteins upregulated in glaucoma, gene ontology analysis points to three major biochemical pathways induced in glaucoma patients compared to controls (Fig 4, see S1 Fig for a GO term relationship diagram). GO analysis revealed a large impact of glaucoma on positive regulation of cholesterol esterification as evidenced by the upregulation of angiotensinogen (AGT), apolipoprotein C-I (APOC1), and apolipoprotein A-IV (APOA4). However, as some patients (two in the glaucoma, one in the control group) received cholesterol-lowering drugs, an influence of systemic medication on this finding cannot completely ruled out. Interestingly, Kliuchnikova and coworkers also identified cholesterol metabolism to be influenced during glaucoma, with apolipoprotein D down-regulated during pseudoexfoliation syndrome [6]. Furthermore, GO analysis revealed that significantly enriched proteins are related to the acute phase response with Scavenger receptor cysteine-rich type 1 protein M130, Alpha-2-antiplasmin, Alpha-1-antichymotrypsin, Inter-alpha-trypsin inhibitor heavy chain H4, Alpha-2-HS-glycoprotein, and Prothrombin upregulated in glaucomatous aqueous humor. Furthermore, proteins including alpha-2-antiplasmin, corticosteroid-binding globulin, vitronectin, alpha-1-antichymotrypsin, inter-alpha-trypsin inhibitor heavy chain H1, thyroxine-binding globulin, inter-alpha-trypsin inhibitor heavy chain H2, AMBP, inter-alpha-trypsin inhibitor heavy chain H4, angiotensinogen, and alpha-2-HS-glycoprotein connected to negatively regulating endopeptidase activity were found to be upregulated.

GO term analysis pointed towards a link between glaucoma and inflammatory processes. Proteins upregulated in the aqueous humor of glaucoma patients were mostly connected to innate immunity. Interestingly, four out of 34 proteins (complement component C1q, C8 beta chain, 9, and V-set and immunoglobulin domain containing protein 4) are connected to the complement system. Furthermore, monocyte/macrophage markers such as CD14, CD163 and chitotriosidase-1 and neutrophile marker neutrophil gelatinase-associated lipocalin were found to be upregulated.

**Fig 5:**
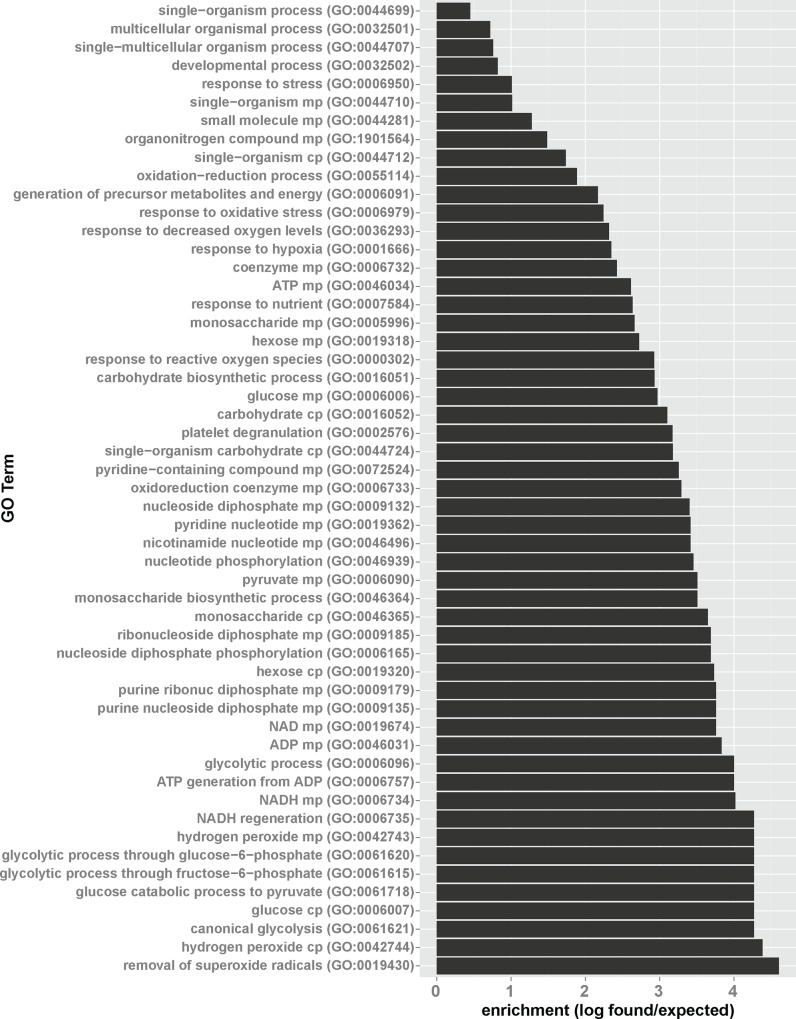
Glaucoma-related downregulation of proteins involved in antioxidant defense, hypoxic response and glycolysis. Proteins found to be downregulated in glaucomatous aqueous humor were analyzed for GO term enrichment. Shown are significantly enriched GO terms (p-Value < 0.01 with Bonferroni correction for multiple comparisons) with enrichment defined as number of proteins found/number of proteins expected based on the human reference genome.

For proteins downregulated in glaucoma, gene ontology enrichment points to a strong suppression of proteins involved in metabolic processes and stress response (Fig 5, see S2 Fig for a GO term relationship diagram). Downregulation of superoxide and hydrogen peroxide-detoxifying proteins such as peroxiredoxin-1, -2 and -6, as well as catalase points to impaired antioxidant defense in glaucomatous aqueous humor. Also, decreased levels of, pyruvate kinase, L-lactate dehydrogenase A chain, ubiquitin-60S ribosomal protein L40, transforming growth factor beta-2, aldehyde dehydrogenase, and pleiotrophin point to lower capacity of glaucoma patients to react to hypoxic stress conditions. On the metabolic side, decreased levels of pyruvate kinase, alpha-enolase, triose phosphate isomerase, phosphoglycerate kinase 1, fructose-bisphosphate aldolase A are indicative for a lower glycolytic capacity of glaucoma patients compared to controls.

As glaucoma is considered a neurodegenerative disease it is reasonable to assume that neuroprotective mechanisms are impacted. Thus, we further analyzed the set of up- and downregulated proteins for a potential connection to neuronal physiology. As shown in Table 1, only downregulated proteins show connection to neuronal physiology while upregulated proteins are not annotated with a function connected to neuronal cells. Of interest in this context is also the observed suppression of antioxidant defense capacity as evidenced by the downregulation of peroxireduxin 2 and 6 as well as superoxide dismutase. Neuronal cells are especially susceptible to increased levels of oxidative stress through reactive oxygens species (ROS) and it is conceivable that the decrease in the antioxidant defense in the aqueous humor reflects the increase oxidative to retinal ganglion cells associated with glaucoma [7].

**Table 1:** Proteins downregulated in glaucomatous aqueous humor are connected to neuronal function.

Gene	Regulated in glaucoma	description
**HSPB1**	down	Connected to neurodegenerative disorder Charcot-Marie-Tooth 2F and distal hereditary motor neuropathy [8, 9]
**PGK1**	down	Loss connected to neurological impairment [10]
**PEBP1**	down	Neuronal development [11]
**SERPINI1 (Neuroserpin)**	down	Axonal growth, synaptic plasticity [12]
**Neurotrimin**	down	Neurite outgrowth and cohesion [13]
**Testican-1, 2, 3**	down	Neurogenesis [14, 15]

The NCBI gene database was searched for annotations connected to neuronal function or disease.

## Discussion

The results of this study suggest an association of changes in the aqueous humor proteome of glaucoma patients compared to a non-glaucomatous control group. Three major processes are impacted in the aqueous humor by glaucoma, namely, glycolysis, processes related to neuronal function, as well as innate immunity. While these processes are clearly differentially regulated in both groups, the influence of multiple medications received by both groups might constitute a confounding factor. This is a concern for the anti-glaucoma medication which was only received by the glaucoma group and not the control group. However, systemic medication received by these patients, e.g. bisoprolol and cilazapril show to influence ocular blood flow [16] and therefore potentially influencing the aqueous humor proteome, are less of a concern as both groups received a variety of different drugs making systemic drug-related bias unlikely.

The significant downregulation of glycolytic enzymes in the aqueous humor of glaucoma patients points to disturbed metabolic processes. It is to expect that due to the death of retinal ganglion cells and the loss of vision the energy requirements of the eye change. And in support of this notion, carbohydrate metabolism was found to be disturbed with enzymes of the Krebs cycle diminished in the trabecular meshwork of glaucomatous eyes [17]. Metabolic enzymes are intracellular proteins and their presence in the extracellular milieu of the aqueous humor is due to cellular leakage leading to a certain steady state concentration of these proteins. The observed decrease of metabolic enzymes does most likely not reflect decreased leakage of such proteins from dying retinal ganglion cells as the loss of these cells occurred already in the past. Thus, the observed suppression of glycolytic enzymes might be due to decreased metabolic flux following suppression of the Krebs cycle inside the cells contributing to the aqueous humor proteome.

Not only glycolytic enzymes but also proteins involved in neuronal function were downregulated in glaucomatous aqueous humor. As glaucoma is a neurodegenerative disorder, the loss of proteins involved in neuronal physiology in the aqueous humor likely reflects the demise of retinal ganglion cells. For example, the lower steady-state level of neurotrimin, a neuron-neuron adhesion molecule, would be a consequence of the lowered amount of retinal ganglion cells producing this protein. And indeed, neurotrimin is highly expressed among other regions in the neuroretina [13], consistent with this interpretation. Furthermore, in an antibody microarray-based study comparing patients undergoing glaucoma or cataract surgery, changes to proteins involved in neuronal survival and function were identified [18].

In contrast to glycolytic enzymes and neuronal proteins, markers for innate immune response are upregulated in the glaucomatous aqueous humor. For example, upregulation CD14 strongly supports increased presence of macrophages. Similarly, upregulation of chitotriosidase-1 secreted by activated macrophages is a clear sign for an innate immunological process in the aqueous humor of glaucoma patients. Thus, there are clear indications for an ongoing infiltration of macrophages and neutrophils into the aqueous humor of glaucoma patients. And indeed, there is growing evidence that glaucoma progression is accompanied by low-grade inflammatory processes [19]. A study comparing the serum proteome of glaucoma patients to healthy controls revealed upregulation of proteins regulating immune and inflammatory-related processes [20]. Thus, our data support the view that glaucoma might have a chronic inflammatory component likely involved in disease progression.

The aqueous humor is produced by the ciliary body and exits the eye via the trabecular meshwork and the uveoscleral outflow pathway [21]. A large proportion of the aqueous humor, however, is directed into the eye cavity which is filled with the vitreous body. Increased intraocular pressure is likely to influence the drainage of aqueous humor via these two pathways, with increased uveoscleral outflow during high tension glaucoma due to increased pressure in the vitreous body. Therefore, also transport of proteins contained in the aqueous humor from the anterior chamber might be impacted by increased intraocular pressure. The best investigated risk factor for glaucoma, so far, is elevated intraocular pressure thought to mainly cause mechanical damage to ganglion cells and axons. Elevated intraocular pressure alone, however, cannot explain glaucoma damage as there are large populations with glaucomatous optic disc appearance and visual field loss despite normal intraocular pressure [22]. Thus, altered protein transport from the aqueous humor into the vitreous body might also contribute to impaired retinal ganglion cell survival under conditions of elevated intraocular pressure.

Neuronal cells are especially susceptible to oxidative stress due to mitochondrial dysfunction [23] and glaucoma as neurodegenerative disorder has an oxidative damage component [24]. Thus, our observation of diminished antioxidant protein levels supports this view of glaucoma as neurodegenerative disorder at least in part promoted by failing antioxidant defense and therefore increased oxidative-damage induced neuronal cell death. Interestingly, antibody microarray-based studies of aqueous humor proteins comparing glaucoma and cataract patients found lower antioxidant along with higher pro-oxidant protein levels in glaucoma patients compared to controls [18, 25]. Further support for our finding of increased oxidative stress in glaucoma patients comes from a study in rats, were increased protein carbonylation was found in glaucomatous compared to control eyes [26].

## Conclusion

Glaucoma is accompanied by profound biochemical changes ranging from imbalanced metabolism, lack of ROS detoxification, low-grade and chronic inflammatory processes which can be detected in the aqueous humor. Thus, proteomic profiling or specific biomarker detection in the aqueous humor may be a way to follow glaucoma progression and potentially to monitor treatment efficacy.

## Material and Methods

### Study Design

The study was designed as a case-control study. All patients were recruited from the department of ophthalmology from the Cantonal Hospital in Aarau in Switzerland. Inclusion criteria were patients with primary open angle glaucoma (POAG) and cataract. Patients with wet AMD, previous intravitreal anti-VEGF treatment, including intraocular steroids were excluded. Patients with any other compromising ocular condition, such as diabetic retinopathy or uveitis, were also considered ineligible for this study. For an overview about patient history, please refer to Table 2. The study was approved by the local ethics committee (Ethikkommission KSA, Prof. Dr. med. M. Bargetzi, F. Adler, PD Dr. med. O. Hilfiker, Dr. med. E. Hindermann, PD. Dr. pharm. St. Mühlebach, HU. Simmen, D. Jerosch) Patients were verbally informed about the study before the surgery was conducted. Participant consent was documented in patient act. This procedure is part of the consent of the local ethical committee.

**Table 2:** Characteristics of POAG patients and control subjects included in this study.

code	gender	Age (years)	IOP	Year of glaucoma diagnosis	OHN thickness (μm)/ peripapillar RNFL (mm^2^)	Visual acuity	Pre-medication	eye-medication
Control 1	f	83	12	-	n.d.	20/50/20/25	Bisoprolol, Amlodipin, Candesartan, Metformin, Gliclazid	-
Control 2	m	57	14	-	n.d.	20/400/20/15	-	-
Control 3	f	85	13	-	82/1,14	20/50/20/25	Hydrochlorothiazid	-
Control 4	f	84	12	-	n.d.	20/50/no data	Pravastatin, Furosemid, AAA	-
Control 5	m	67	13	-	n.d.	20/40/20/15	Aliskiren, Lercanidipin, Omeprazol	-
Average control		75±13	-		-			
Glaucoma 1	f	88	22	2012	61/0,83	20/200/20/30	Simvastatin, Metoprolol Valsartan, ASS, Levothyroxin, Calcium, Esomeprazol, Vit D3, Ginkgo	Timolol, Tafluprost
Glaucoma 2	f	87	18	2008	65/0,65	20/50/20/30	Metoprolol, Candesartan, Pantoprazol, Candesartan, Zolpidem, Ibandronat, Calcium, Pravastatin, Clopidogrel, ASS, Ginkgo	Timolol, Brimonidin, Travoprost
Glaucoma 3	f	55	28	2006	n.d.	20/50/20/100	-	Timolol, Brimonidin, Bimatoprost
Glaucoma 4	m	80	18	2009	68/n.d.	20/40/20/25	-	Timolol
Glaucoma 5	m	84	24	1987	60/n.d.	20/40/20/50	Irbesartan, Nifedipin	Timolol
Average glaucoma		78S±13	-		-			

Shown are age, gender, intraocular pressure (IOP), optic nerve head thickness (OHN) and area of the peripapillar retinal nerve fiber layer (RNFL), visual acuity, as well as non-eye related medication and medication to treat eye-related conditions. All patients underwent cataract surgery during which sampling of the aqueous humor took place.

### Sampling

Sampling of aqueous humor 10 to 200 μl of aqueous humor from 5 patients with glaucoma and 5 patients with cataract was aspirated from the anterior chamber using a 26 gauge needle before the start of surgery and immediately frozen at -70°C.

### Mass Spectrometry and Data Analysis

Protein samples were processed for and analyzed by mass spectrometry at Biognosys, Zurich, Switzerland. In short, urea-denatured proteins were alkylated with iodoacetamide, digested using sequencing grad modified trypsin, purified and analyzed using Biognosys’ proprietary protocol. Peptides (calculated amount 1.5 μg per sample) were injected to an in-house packed C18 column (Magic AQ, 3μm particle size, 200 Å pore size, Michrom; 75μm inner diameter, New Objective) on a Thermo Scientific Easy nLC nano-liquid chromatography system for all mass spectrometric analysis. LC solvents were A: 1% acetonitrile in water with 0.1% FA; B: 3% water in acetonitrile with 0.1% FA. The LC gradient for shotgun analysis was 0–72% solvent B in 120 minutes (non-linear) followed by 72–100%B in 2 minutes and 100% B for 8 minutes (total gradient length was 130 minutes). Column length was 30cm. All mass spectrometric analyses were carried out on a Thermo Scientific Q Exactive mass spectrometer equipped with a standard nano-electrospray source. Full MS for shotgun LC-MS/MS covered the m/z range of 400–1200 with a resolution of 70’000 (AGC target value was 1e6) and was followed by 12 data dependent MS2 scans with a resolution of 17’500 (AGC target value was 5e5). MS2 acquisition precursor isolation width was 2 m/z while normalized collision energy was centered at 25 (10% stepped collision energy) and the default charge state was 2+. In HRM-MS™ mode, full MS covered the m/z range of 400–1220 and all-ion fragmentation (AIF) scan range was 200–1800 m/z. LC-MS/MS datasets were analyzed using the MaxQuant software package v 1.3.0.5 and searches were performed against the UniProt HUMAN database. HRM-MS™ maps were analysed with Spectronaut™ software using the library generated from MaxQuant searches of shotgun runs. The applied false discovery rate cutoff was 0.01.

### GO Term Enrichment

GO term enrichment was performed using the GO enrichment tool at http://geneontology.org/page/go-enrichment-analysis [27, 28]. Further GO term analysis was performed using the QuickGO tool at http://www.ebi.ac.uk/QuickGO/ [29]. A list of all peptides is added to this paper in S1 Table HRMprofiling.

## Supporting Information

S1 Fig Gene ontology analysis for proteins upregulated in the aqueous humor of glaumcoma patients compared to controls. Shown is a QuickGO tool analysis.
(TIF)

S2 Fig Gene ontology analysis for proteins downregulated in the aqueous humor of glaumcoma patients compared to controls. Shown is a QuickGO tool analysis.
(TIF)

S1 Table HRMprofiling original workfile with all Protein descriptions, peptide assays used for profiling, peptides used for profiling and statistics. (XLSX)
