# Supplementary figures and images for: Changes to the Aqueous Humor Proteome during Glaucoma
Source: PLoS One. 2016 Oct 27;11(10):e0165314. doi: 10.1371/journal.pone.0165314 (PMC5082887; doi:10.1371/journal.pone.0165314)

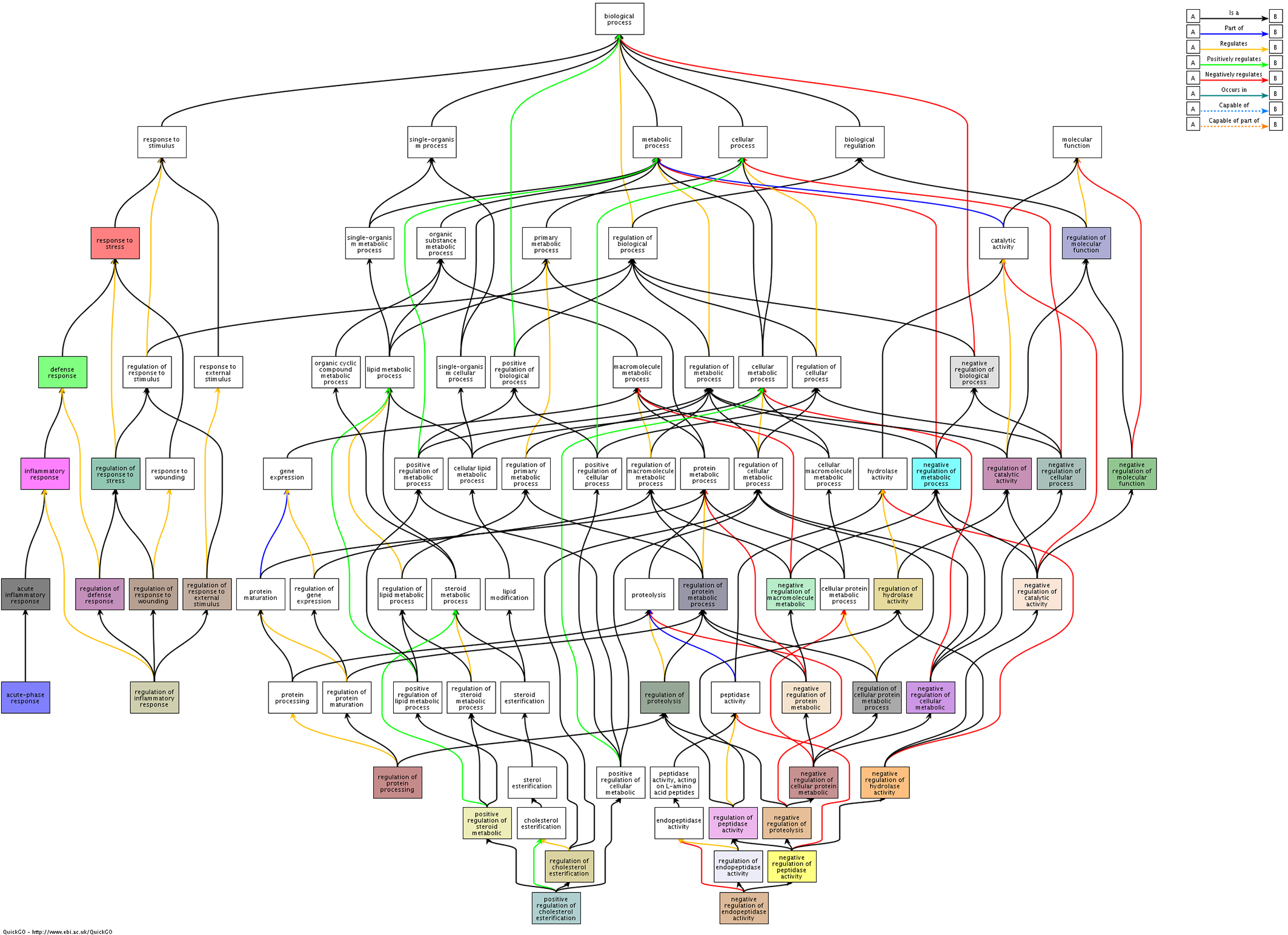

Supplement: S1 Fig — Shown is a QuickGO tool analysis. (TIF) [file pone.0165314.s001.tif]

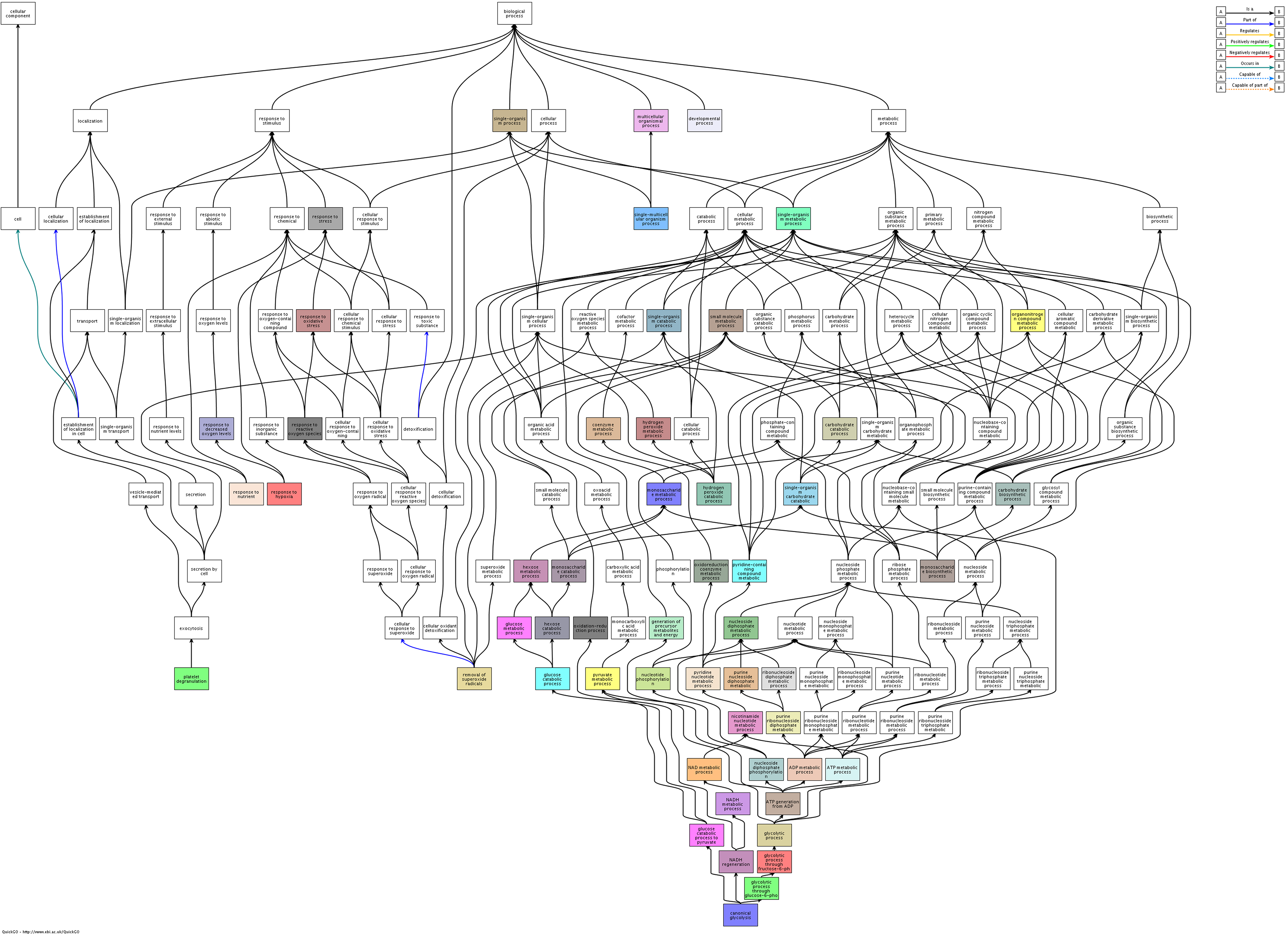

Supplement: S2 Fig — Shown is a QuickGO tool analysis. (TIF) [file pone.0165314.s002.tif]
